# Silicon enhances plant resistance to *Fusarium* wilt by promoting antioxidant potential and photosynthetic capacity in cucumber (*Cucumis sativus* L.)

**DOI:** 10.3389/fpls.2022.1011859

**Published:** 2022-10-13

**Authors:** Shuangsheng Sun, Zhengkun Yang, Zhiyu Song, Nannan Wang, Ning Guo, Jinghan Niu, Airong Liu, Bing Bai, Golam Jalal Ahammed, Shuangchen Chen

**Affiliations:** ^1^ College of Horticulture and Plant Protection, Henan University of Science and Technology, Luoyang, China; ^2^ Wageningen Seed Science Centre, Laboratory of Plant Physiology, Wageningen University, Wageningen, Netherlands; ^3^ Henan International Joint Laboratory of Stress Resistance Regulation and Safe Production of Protected Vegetables, Luoyang, China; ^4^ Henan Engineering Technology Research Center for Horticultural Crop safety and Disease Control, Luoyang, China

**Keywords:** silicon, cucumber, *Fusarium oxysporum*, photosynthesis, antioxidant system

## Abstract

*Fusarium* wilt, caused by *Fusarium oxysporum* f. sp. *cucumerinum* (Fo), is a severe soil-borne disease affecting cucumber production worldwide, particularly under monocropping in greenhouses. Silicon (Si) plays an important role in improving the resistance of crops to *Fusarium* wilt, but the underlying mechanism is largely unclear. Here, an *in vitro* study showed that 3 mmol·l^-1^ Si had the best inhibitory effect on the mycelial growth of *F. oxysporum* in potato dextrose agar (PDA) culture for 7 days. Subsequently, the occurrence of cucumber wilt disease and its mechanisms were investigated upon treatments with exogenous silicon under soil culture. The plant height, stem diameter, root length, and root activity under Si+Fo treatment increased significantly by 39.53%, 94.87%, 74.32%, and 95.11% compared with Fo only. Importantly, the control efficiency of Si+Fo was 69.31% compared with that of Fo treatment. Compared with Fo, the activities of peroxidase (POD), catalase (CAT), and ascorbate peroxidase (APX) significantly increased by 148.92%, 26.47%, and 58.54%, while the contents of H_2_O_2_, $O_{2}^{\cdot -}$ , and malondialdehyde (MDA) notably decreased by 21.67%, 59.67%, and 38.701%, respectively, in roots of cucumber plants treated with Si + Fo. Compared with Fo treatment, the net photosynthesis rate (Pn), stomatal conductance (Gs), transpiration rate (Tr), maximum RuBisCO carboxylation rates (*V*cmax), maximum RuBP regeneration rates (*J*max), and activities of ribulose-1,5-bisphosphate carboxylase (RuBisCO), fructose-1,6-bisphosphatase (FBPase), and glyceraldehyde-3-phosphate dehydrogenase (GAPDH) and the expression of *FBPA*, *TPI*, *SBPase*, and *FBPase* in Si+Fo treatment increased significantly. Furthermore, Si alleviated stomatal closure and enhanced endogenous silicon content compared with only Fo inoculation. The study results suggest that exogenous silicon application improves cucumber resistance to *Fusarium* wilt by stimulating the antioxidant system, photosynthetic capacity, and stomatal movement in cucumber leaves. This study brings new insights into the potential of Si application in boosting cucumber resistance against *Fusarium* wilt with a bright prospect for Si use in cucumber production under greenhouse conditions.

## Introduction

Cucumber (*Cucumis sativus* L.) is currently one of the vastly cultivated and economically beneficial vegetables of the Cucurbitaceae family in the world. However, its production is increasingly being affected by fungal diseases, especially *Fusarium* wilt, one of the three most serious fungal diseases of cucumber plants (Raza et al., 2017). *Fusarium* wilt, caused by *F. oxysporum*, is a destructive soil-borne fungal disease (Zhai et al., 2021). The cucumber-specific type of *F. oxysporum* can colonize the vascular bundles of cucumber rhizomes, preventing the transportation of nutrients and water (Zhou et al., 2017), resulting in the wilting of aboveground parts of cucumber plants (Sun et al., 2021). *Fusarium* wilt significantly reduces the photosynthesis capacity and photosynthetic enzyme activity and can also cause a 15%–50% reduction in cucumber yield (Ahammed et al., 2020). Since pesticides are still used to prevent and control cucumber *Fusarium* wilt, the pesticide residue has brought a great threat to human health and environmental sustainability (Jain et al., 2022). Therefore, it is a hot topic in recent years to find a pollution-free and effective method to prevent cucumber wilt.

Silicon (Si) is a tetravalent chemical element, which is mainly taken by plants in the form of monosilicic acid (H_4_SiO_4_) from the soil (Shivaraj et al., 2022). Si is widely recognized as an element beneficial to plant growth and biomass production. Studies have demonstrated that exogenous Si treatment can significantly improve the yield of rice, tomato, and other crops (Hoffmann et al., 2020; Chaiwong and Prom-u-thai, 2022). Si can thicken the main stems of plants, improve photosynthesis, and enhance the development of vascular bundles involved in the uptake and transport of nutrients (Pavlovic et al., 2021). Moreover, Si can form a “keratin-silicon double-layer” physical barrier on the plant leaf surface to resist insects and fungi (Yoshid, 1965). Si is also known to effectively mitigate various abiotic stresses such as heavy metal toxicities, salinity, drought, chilling, and freezing stresses (Savvas and Ntatsi, 2015). The application of Si increased the salt tolerance of tomato plants by enhancing substomatal CO_2_, net photosynthetic rate, photosynthetic water use efficiency, and mesophyll conductance (Haghighi and Pessarakli, 2013). Si could alleviate adverse effects of salt stress by reducing the Na^+^ concentration and boosting antioxidant enzyme activities in *Glycyrrhiza uralensis*, and these alleviating effects were dependent on Si concentrations and the time of Si treatment (Li et al., 2016).

It is reported that Si addition to soil can significantly enhance the resistance of plants against disease and increase plant immune response. Supplying Si to tomato seedlings can reduce the disease severity of *Fusarium* crown and root rot in tomato; the increase in the Si content of roots was significantly correlated with the reduction of disease severity of root, crown, and stem (Huang et al., 2011). Furthermore, Si alleviated soil-borne disease stress by adjusting soil microbial composition and diversity, in which Si-added soil harbored a lower proportion of *Pseudomonas*, *Fusarium*, and *Faecalibacterium* (Lin et al., 2020). It was reported that exogenous Si and silicate salts significantly stimulated systemic defense enzymes in onion and garlic plants and decreased the incidence of white rot disease (Elshahawy et al., 2021). Si treatment could increase the late blight resistance of potato plants by increasing ethylene and jasmonic acid metabolism in both detached leaves and intact plants (Xue et al., 2021).

Application of Si is a preventive strategy against many soil-borne fungal diseases, such as *Pythium* damping off in cucumber (Al Sadi et al., 2010), *Fusarium* wilt in banana plants (Fortunato et al., 2014), *F. oxysporum* on cotton (Whan et al., 2016), and black pepper plants (D'Addazio et al., 2020). Silicon can induce a higher lignin concentration in moderately resistant cultivars than that in less resistant cultivars. However, the contribution of antioxidant potential and the photosynthetic capacity of cucumber to Si-mediated *Fusarium* wilt stress alleviation remain unclear.

In the current study, we examined the effects of sodium silicate (Na_2_SiO_3_·9H_2_O) treatment on the occurrence of cucumber wilt disease and its mechanisms. Our results revealed that Si-induced enhanced resistance to *Fusarium* wilt was closely related to the stimulation of photosynthetic capacity and antioxidant system of cucumber leaves. The study suggested the great potential of Si as a means of biological control of *F. oxysporum* in cucumber plants that could not only reduce losses caused by *Fusarium* wilt but also minimize the use of chemical fungicides.

## Materials and methods

### Plant materials, fungal strains, and treatments

In the present study, cucumber (*Cucumis sativus* L. cv. Jinyan No. 4) which is susceptible to *Fusarium* wilt was used as plant material (Liu et al., 2010). *F. oxysporum* f. sp. *cucumerinum* isolates were cultured on Komada’s *Fusarium*-selective medium (Isack et al., 2014) and confirmed on the basis of microscopic studies of the shape and size of macro- and micro-conidia (Booth, 1971). Analytically pure sodium silicate (Na_2_SiO_3_·9H_2_O) was purchased from Sangon Biotech Co., Ltd. (Shanghai, China). The tested soil was imported peat soil from Germany K brand, with soil organic matter content of 25.25 g/kg, alkali hydrolyzable nitrogen content of 66.8 mg/kg, available phosphorus content of 12.37 mg/kg, available potassium content of 96.53 mg/kg, and pH of 6.50. Seeds of cucumber were surface sterilized with 0.1% potassium permanganate solution and germinated in peat: vermiculite = 2:1 (v:v).

### Screening of silicon concentrations

The *in vitro* plate culture method was used to evenly smear the sterilized Na_2_SiO_3_·9H_2_O solution with concentrations of 1, 2, 3, 4, and 5 mmol L^-1^ Si on the surface of potato dextrose agar (PDA) medium. *F. oxysporum* f. sp. *cucumerinum* was inoculated on the culture medium with a 5-mm-diameter punch. At the same time, the plate coated with distilled water and inoculated with *Fusarium* was cultured as the reference control. Each treatment was repeated three times. The mycelial growth diameter was observed and recorded for 5–7 days. The inhibition rate was calculated, and the differences were analyzed to select the most suitable concentration of Si.

### Experimental design

The experiment was performed in a greenhouse at Henan University of Science and Technology, Luoyang, China (longitude 112°26′E and latitude 34°40′N), with the following growth conditions: 28°C/20°C (day/night) temperatures, 85% relative humidity, and a 12h photoperiod with an average photosynthetic flux of 600 μmol m^-2^ s^-1^. The cucumber seedlings at the four-leaf stage were irrigated with 100 ml (per pot) of 3 mmol·l^-1^ Na_2_SiO_3_·9H_2_O, and the treatment was repeated every 7 days (in total four times). The control was irrigated with 100 ml of sterilized water. Then, one-half of Si-treated seedlings and one-half of water-inoculated seedlings were inoculated with *F. oxysporum* by adding a conidial suspension of *F. oxysporum* into the nutrient solutions to achieve a final concentration of 10^6^ conidia per milliliter. The details of the preparation of *F. oxysporum* suspension and treatment have been described in our previous study (Ahammed et al., 2020). To determine the changes in plant height, stem diameter and root length were measured at 0 day (the day when inoculation was performed) and at 9 days after inoculation with *F. oxysporum*.

### Gas exchange and chlorophyll fluorescence measurements

Gas exchange analysis including the net photosynthetic rate (Pn), stomatal conductance (Gs), intercellular carbon dioxide concentration (Ci), and transpiration rate (Tr) of leaves was conducted using an infrared gas analyzer-based portable photosynthesis system (LI-6400; LI-COR, Lincoln, NE, USA) on the fourth leaf of each plant. *A*sat was measured at an ambient CO_2_ concentration of 360 µmol mol^-1^ and saturating PPFD (1,000 µmol m^-2^ s^-1^) with a leaf temperature of 25 ± 1.5°C and air relative humidity of 80%–90%. Assimilation versus intercellular CO_2_ concentration (*A*/*C*i) curves were measured based on the method of von Caemmerer and Farquhar (1981). The maximum RuBisCO carboxylation rates (*V*cmax) and maximum RuBP regeneration rates (*J*max) were estimated from the *A*/Ci curves according to Ethier and Livingston (2004).

### Determination of enzyme activity involved in the Calvin cycle

Ribulose-1,5-bis-phosphate (RuBP) carboxylase/oxygenase (RuBisCO) activity was measured spectrophotometrically by coupling 3-phosphoglyceric acid formation with NADH oxidation at 25°C, following the method described by Lilley and Walker (1974). FBPase activity was determined by monitoring the increase in A340 using an extinction coefficient of 6.2 mM^-1^ cm^-1^ (Scheibe et al., 1986; Zhou et al., 2007). The initial activity was assayed immediately after homogenization. Total activity was assayed on aliquots of enzyme extract incubated for 20 min with 100 mM dithiothreitol, 2 mM Fru-1,6-bisP, 10 mM MgCl_2_, and 0.1 M HEPES-NaOH (pH 8.0). The measurement of GAPDH activity was according to the method of Piattoni et al. (2013).

### Estimation of antioxidant enzyme activity

The 0.5g sample was ground to a homogenate with 3 ml precooled potassium phosphate buffer (50 mol l^-1^, pH = 7.0) in an ice bath followed by centrifugation for 20 min at 10,000 g at 4°C. Extracting solution was used to determine the superoxide dismutase (SOD), guaiacol peroxidase (G-POD), catalase (CAT), and ascorbate peroxidase (APX) activity. SOD activity was examined by measuring the ability of SOD to inhibit the photochemical reduction of nitro blue tetrazolium at 560 nm (Aebi, 1984). CAT activity was determined *via* the reduction at 240 nm for H_2_O_2_ extinction (Giannakoula et al., 2010). APX activity was measured based on the rate of AsA oxidation. A 0.1-ml supernatant was mixed with 1 ml extracting solution (50 mol l^-1^ potassium phosphate with pH = 7.0, 750 µmol l^-1^ AsA, and 100 mol l^-1^ H_2_O_2_). The absorbance at 290 nm was measured at every 15-s interval for 3 min (Durner and Klessig, 1995). The enzyme extracts of guaiacol peroxidase (G-POD) were isolated following the method of Lamikanra and Watson (2001). G-POD activity (EC 1.11.1.7) was determined by the enhancement in absorbance at 470 nm (ϵ, 26.6 mM^-1^ cm^-1^) which resulted from the oxidation of guaiacol (Cakmak and Marschner, 1992).

### Biochemical quantification of reactive oxygen species and malondialdehyde levels

H_2_O_2_ content in roots was determined spectrophotometrically by a peroxidase assay according to Willekens et al. (1997). The $O_{2}^{\cdot -}$ production rate was measured according to the method of Elstner and Heupel (Elstner and Heupel, 1976). The level of lipid peroxidation in roots was determined by quantifying the malondialdehyde (MDA) equivalents using 2-thiobarbituric acid (TBA) as described by Hodges et al. (1999).

### RNA extraction and qRT-PCR for gene expression analysis

Total RNA was extracted from root tissues using TRIzol reagent (Sangon, China) at 1 dpi with FO according to the manufacturer’s instructions. Genomic DNA was removed by DNase treatment (BBI, Canada). One milligram of total RNA was reverse-transcribed using ReverTra Ace qPCR RT Kit (Toyobo, Japan) according to the manufacturer’s instruction. The iCycler iQ™ real-time PCR detection system (Bio-Rad, Hercules, CA, USA) was used for quantitative real-time PCR. Twenty-five-microliter reaction mixtures consisted of 12.5 µl SYBR Green PCR Master Mix (Takara, Japan), 1 µl of diluted cDNA, and 0.2 µM of forward and reverse primers. The conditions for PCR cycling were as follows: 95°C for 3 min and 40 cycles of 95°C for 10 s 58°C for 45 s. The gene-specific primers used for the amplification were determined on the basis of gene or EST sequences and are listed in **Supplementary Table S1**. mRNA levels were quantified according to the method of Livak and Schmittgen (2001). To obtain a Δ*C*t value, the threshold cycle (*C*t) value of *actin* was subtracted from that value of the gene of interest.

### Statistical analysis

All data presented are mean values of three repetitions of each treatment. Data were statistically analyzed using analysis of variance (AVONA) and expressed as mean ± standard deviation (SD). We used Tukey’s least significant difference test at 5% for multiple pairwise comparisons.

## Results

### Screening of optimal silicon concentrations

Five concentration gradients of Na_2_SiO_3_·9H_2_O were used in the *in vitro* test. After the culture of *F. oxysporum* f. sp. *cucumerinum* on the PDA plate for 7 days, the mycelial growth of *Fusarium* on the control plate reached close to the full plate, while the mycelial growth in the plate coated with Na_2_SiO_3_·9H_2_O was significantly inhibited.

**Figure 1:**
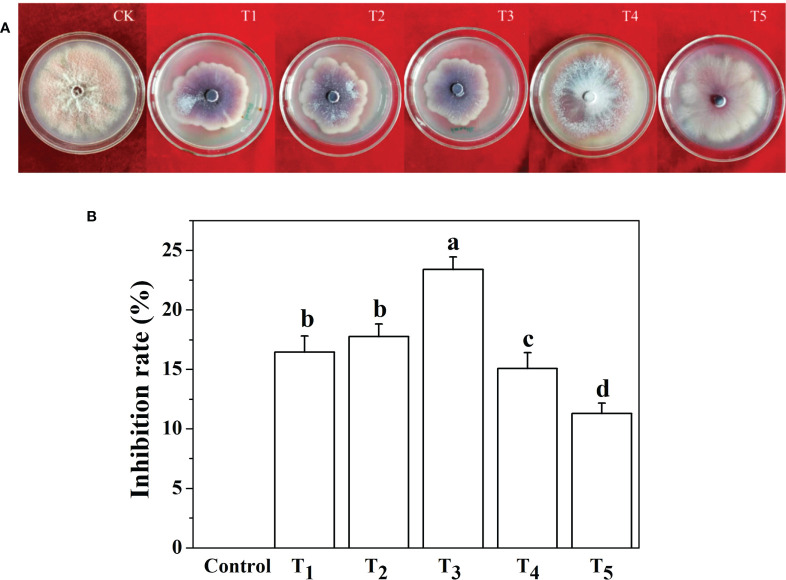
Screening of optimal silicon (Si) concentrations against *Fusarium oxysporum* (f) sp. *cucumerinum* fungi. **(A)** Photographs of *in vitro* culture of *Fusarium* fungi in five different silicon concentrations on PDA plates for 7 days. **(B)** Inhibition rate of five silicon concentrations on mycelial growth of *Fusarium* when cultured for 7 days. CK or control, 0 mmol·l^-1^ Si; T_1_, 1 mmol·l^-1^ Si; T_2_, 2 mmol·l^-1^ Si; T_3_, 3 mmol·l^-1^ Si; T_4_, 4 mmol·l^-1^ Si; and T_5_, 5 mmol·l^-1^ Si. Means denoted by the different lowercase letters indicate a significant difference according to Tukey’s test (*P* ≤ 0.05).

The five concentrations of Si tested all had certain antifungal effects on *Fusarium* growth *in vitro*. After 7 days of PDA culture, 3 mmol·l^-1^ Na_2_SiO_3_·9H_2_O showed the best inhibitory effect on *Fusarium* growth, and the inhibition rate reached 23.4% (**Figure 1**). Five millimoles per liter of Na_2_SiO_3_·9H_2_O had the least inhibitory effect on *Fusarium* growth. Therefore, 3 mmol·l^-1^ Na_2_SiO_3_·9H_2_O was selected as the optimal Si concentration for the following test.

### Silicon enhances cucumber growth and resistance to *F. oxysporum*

**Figure 2:**
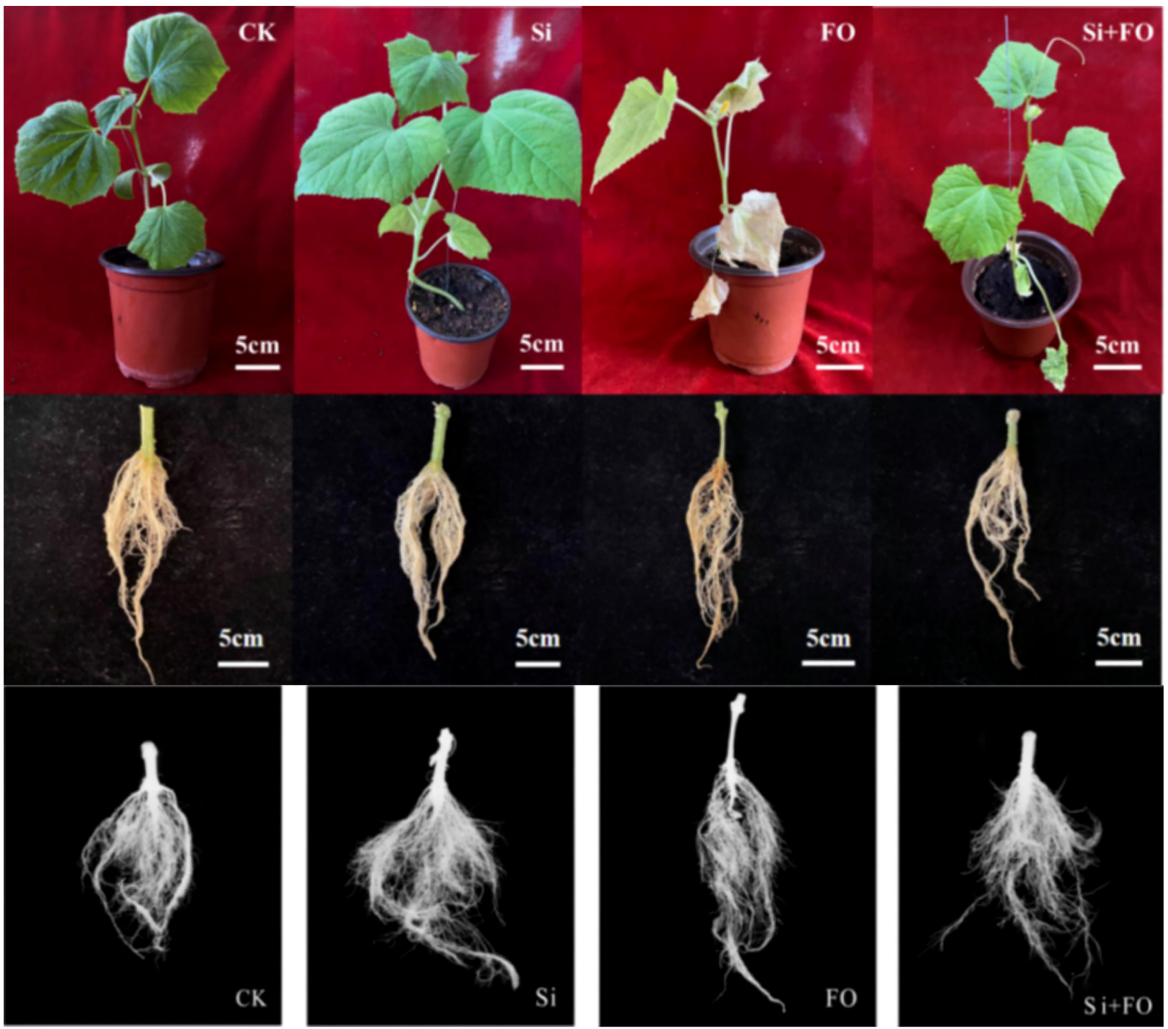
Phenotypes of cucumber plants under different treatments. Upper panels: whole plant phenotype, middle panels: root phenotype, and lower panels: images of scanned roots under different treatments. CK or control: plants were irrigated with sterilized water; Si: plants were irrigated with exogenous silicon (3 mmol·l^-1^ Si); FO: plants were only inoculated with *Fusarium*; Si+FO: plants were irrigated with exogenous silicon followed by inoculation with *Fusarium*.

The plants treated with exogenous Si were more vigorous than other treatments, and the plants have more roots. However, the leaves of only Fo-treated plants showed leaf withering, and the roots were gradually rotted. However, with Si application to the roots (i.e., Si + Fo), the withering symptom of cucumber leaves and the rotted symptom of cucumber roots were significantly alleviated (**Figure 2**).

Compared with the control, the plant height, stem diameter, root length, and root activity of Fo treatment decreased by 39.86%, 54.47%, 49.32%, and 60.53%, respectively. However, the plant height, stem diameter, root length, and root activity of Si+Fo treatment increased significantly by 39.53%, 94.87%, 74.32%, and 95.11%, respectively, compared with Fo only (**Table 1**). Additionally, exogenous Si application remarkably improved various root traits (**Table 2**) Importantly, exogenous Si application significantly decreased disease index and the control efficiency of Si+Fo was 69.31% compared with that of Fo treatment (**Table 3**).

**Table 1:** Effects of exogenous silicon on root length, plant height, and stem diameter of cucumber as influenced by *Fusarium* wilt.

Treatments	Height (cm)	Stem diameter (cm)	Root length (cm)	Root activities (mg g^-1^ h^-1^ FW)
Control	14.3 ± 0.4^b^	0.257 ± 0.007^b^	14.6 ± 0.5^b^	0.78 ± 0.04^b^
Si	18.1 ± 1.3^a^	0.335 ± 0.016^a^	18.3 ± 1.6^a^	0.93 ± 0.05^a^
FO	8.6 ± 1.8^d^	0.117 ± 0.001^d^	7.4 ± 0.2^c^	0.32 ± 0.02^c^
Si+FO	12.0 ± 0.5^c^	0.228 ± 0.008^c^	12.9 ± 0.4^b^	0.65 ± 0.04^b^

Means (± standard deviation) denoted by the different lowercase letters in each column are significantly different according to Tukey’s test (P ≤ 0.05).

**Table 2:** Effects of exogenous silicon application on root traits of cucumber plants as influenced by *Fusarium* wilt.

Treatments	Control	Si	FO	Si+FO
Length (cm)	742.56 ± 25.31^b^	924.61 ± 78.23^a^	573.47 ± 41.23^d^	664.54 ± 50.13^c^
SA (cm^2^)	143.46 ± 8.63^b^	178.84 ± 14.05^a^	118.85 ± 10.25^c^	134.94 ± 12.08^c^
PA (cm^2^)	44.32 ± 3.07^b^	57.25 ± 4.06^a^	36.188 ± 2.68^c^	43.31 ± 3.65^b^
VOL (cm^3^)	2.41 ± 0.21^b^	2.76 ± 0.19^a^	1.81 ± 0.23^d^	2.02 ± 0.18^c^
AvgD (mm)	0.65 ± 0.04^b^	0.72 ± 0.06^a^	0.56 ± 0.04^d^	0.62 ± 0.05^c^
Ntips (pcs)	1837 ± 163^b^	2019 ± 156^a^	1437 ± 109^d^	1595 ± 128^c^
Nforks (pcs)	7859 ± 418^b^	11060 ± 856^a^	6489 ± 492^c^	7184 ± 597^bc^

Means (± standard deviation) denoted by the different lowercase letters in each column are significantly different according to Tukey’s test (P ≤ 0.05). Length: root length; SA: root surface area; PA: total root projected unit area; VOL: overall volume; AvgD: root average diameter; Ntips: total number of root tips; Nforks: number of branches.

**Table 3:** Control effect of exogenous silicon on *Fusarium* wilt in cucumber plants.

Treatments	Disease index	Control effect
FO	89.67 ± 6.52^a^	–
Si+FO	27.52 ± 1.59^b^	69.31 ± 3.46%

Means (± standard deviation) denoted by the different lowercase letters in each column are significantly different according to Tukey’s test (P ≤ 0.05).

### Silicon alleviates *Fusarium* wilt-induced oxidative stress

**Figure 3:**
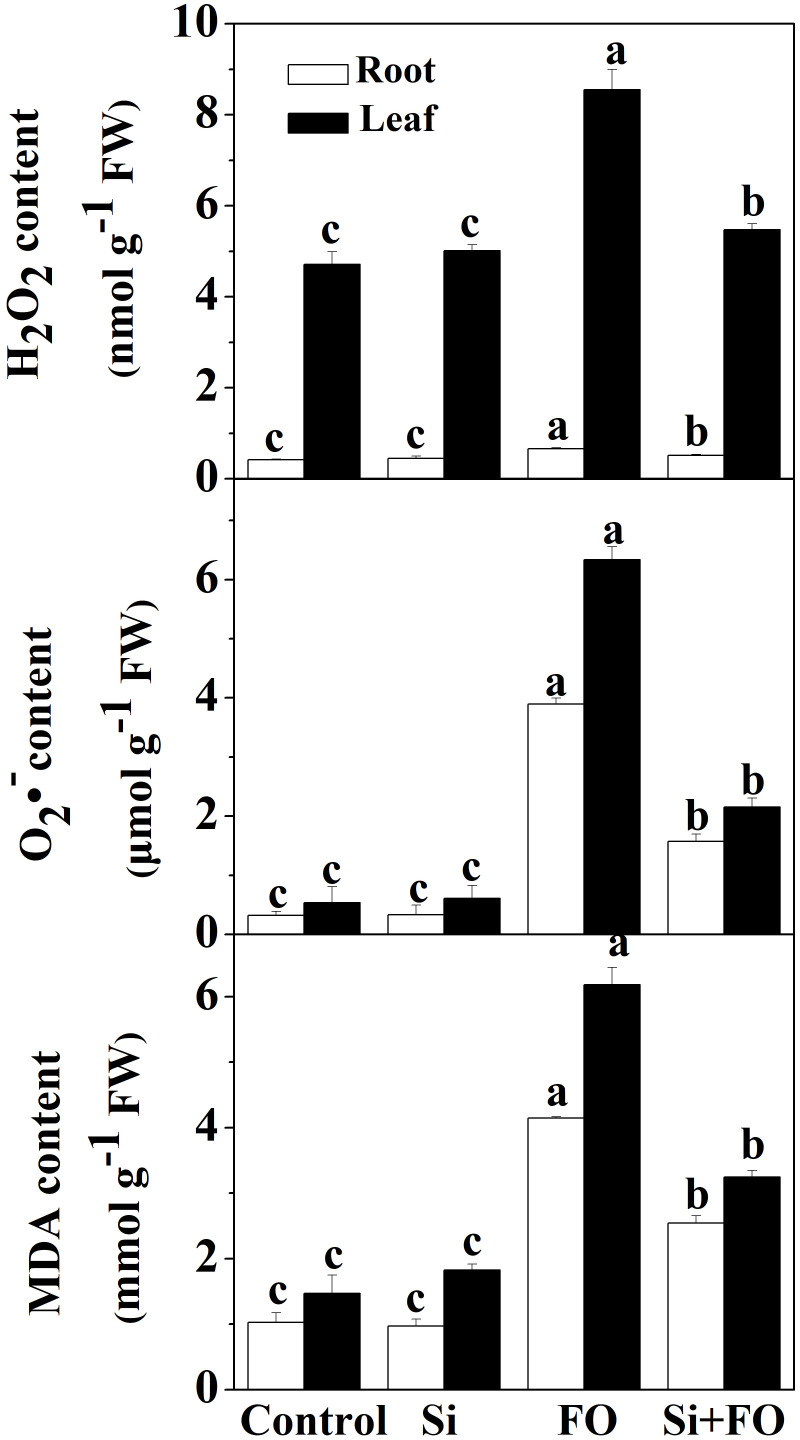
Effects of exogenous silicon application on the contents of H_2_O_2_, $O_{2}^{\cdot -}$ , and MDA in cucumber roots. Means denoted by the different lowercase letters are significantly different according to Tukey’s test (*P* ≤ 0.05); the mean represents the average of three replicates ± standard deviation (SD). Control: plants were irrigated with sterilized water; Si: plants were irrigated with exogenous silicon (3 mmol·l^-1^ Si); FO: plants were only inoculated with *Fusarium*; Si+FO: plants were irrigated with exogenous silicon followed by inoculation with *Fusarium*.

To assess the effect of *Fusarium* inoculation on oxidative stress markers, the contents of hydrogen peroxide (H_2_O_2_), superoxide anion ( $O_{2}^{\cdot -}$ ), and malondialdehyde (MDA) in the roots and leaves were determined. Compared with the control, the H_2_O_2_, $O_{2}^{\cdot -}$ , and MDA contents in roots under Fo treatment increased by 0.56, 11.06, and 0.39 times, respectively, and the H_2_O_2_, $O_{2}^{\cdot -}$ , and MDA contents in leaves increased by 0.82, 10.85, and 3.21 times, respectively (**Figure 3**). However, Si application significantly decreased *Fusarium* wilt-induced reactive oxygen species (ROS) accumulation in cucumber roots and leaves. Compared with Fo treatment, the contents of H_2_O_2_, $O_{2}^{\cdot -}$ , and MDA in roots of Si+Fo treatment decreased by 21.67%, 59.67%, and 38.70%, while those in leaves decreased by 35.98%, 66.02%, and 47.51%, respectively.

### Exogenous silicon activates antioxidant enzymes

**Figure 4:**
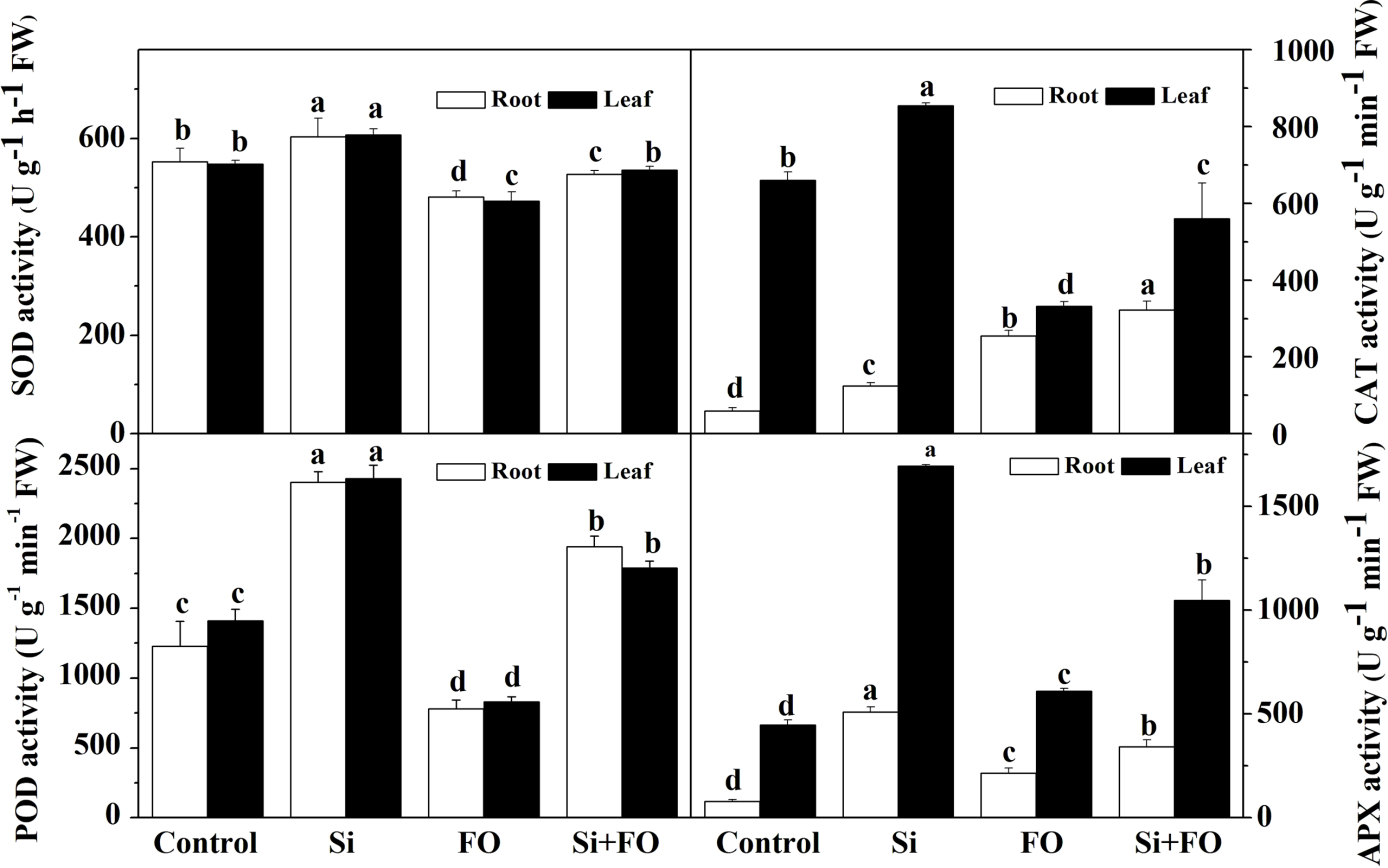
Effects of exogenous silicon application on the activities of POD, CAT, SOD, and APX in cucumber roots. Means denoted by the different lowercase letters are significantly different according to Tukey’s test (*P* ≤ 0.05); the mean represents the average of three replicates ± standard deviation (SD). Control: plants were irrigated with sterilized water; Si: plants were irrigated with exogenous silicon (3 mmol·l^-1^ Si); FO: plants were only inoculated with *Fusarium*; Si+FO: plants were irrigated with exogenous silicon followed by inoculation with *Fusarium.*.

As shown in **Figure 4**, the activities of antioxidant enzymes, such as POD, CAT, SOD, and APX, in roots of cucumber treated with Si significantly increased by 95.82%, 108.33%, 9.16%, and 546.67%, and in the meantime, those in leaves increased by 72.04%, 29.56%, 10.77%, and 278.82% compared with the control, respectively. However, *Fusarium* decreased POD and SOD activities in roots significantly by 36.41% and 12.98%, respectively, compared with the control. Compared with Fo, the activities of POD and APX in cucumber plants treated with Si + Fo significantly increased by 148.92% and 115.26% in roots and 58.54% and 71.55% in leaves, respectively.

### Silicon enhances photosynthesis rate, *J*max, and *V*cmax

**Figure 5:**
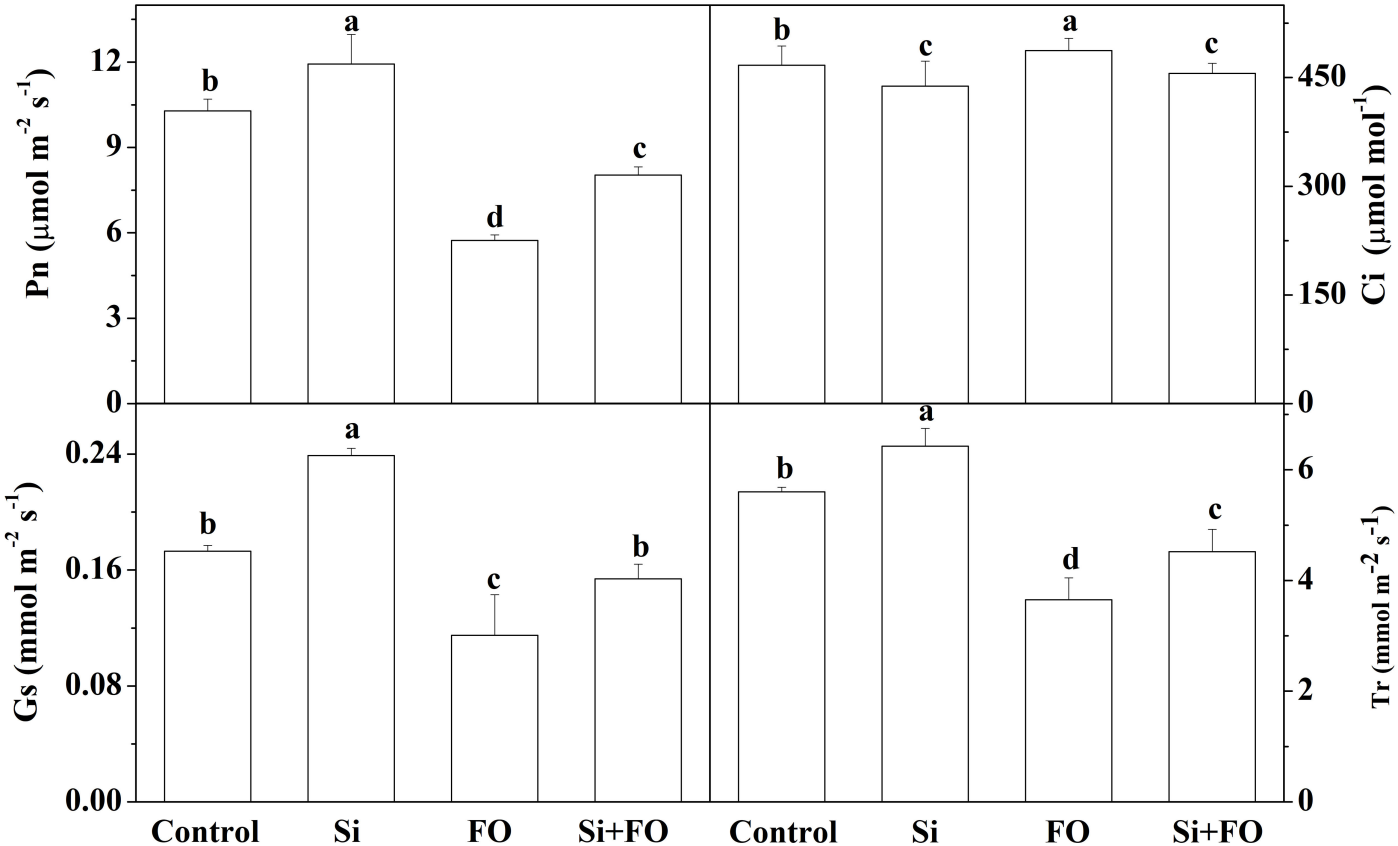
Effects of exogenous silicon application on Pn, Ci, Gs, and Tr in cucumber leaves. Means denoted by the different lowercase letters are significantly different according to Tukey’s test (*P* ≤ 0.05); the mean represents the average of three replicates ± standard deviation (SD). Control: plants were irrigated with sterilized water; Si: plants were irrigated with exogenous silicon (3 mmol·l^-1^ Si); FO: plants were only inoculated with *Fusarium*; Si+FO: plants were irrigated with exogenous silicon followed by inoculation with *Fusarium.*.

Compared with the control, the net photosynthetic rate (Pn), stomatal conductance (Gs), and transpiration rate (Tr) of cucumber leaves in the Si treatment group were significantly increased by 16.05%, 38.15%, and 11.49%, respectively, while the intercellular carbon dioxide concentration (Ci) was significantly reduced by 6.62%. Inoculation with Fo decreased Pn, Gs, and Tr compared with control. Compared with Fo treatment, Pn, Gs, and Tr increased by 40.02%, 28.70%, and 23.84%, while Ci decreased by 6.92% in Si+Fo treatment. The results showed that exogenous Si application could enhance the photosynthetic capacity of cucumber leaves (**Figure 5**).

**Figure 6:**
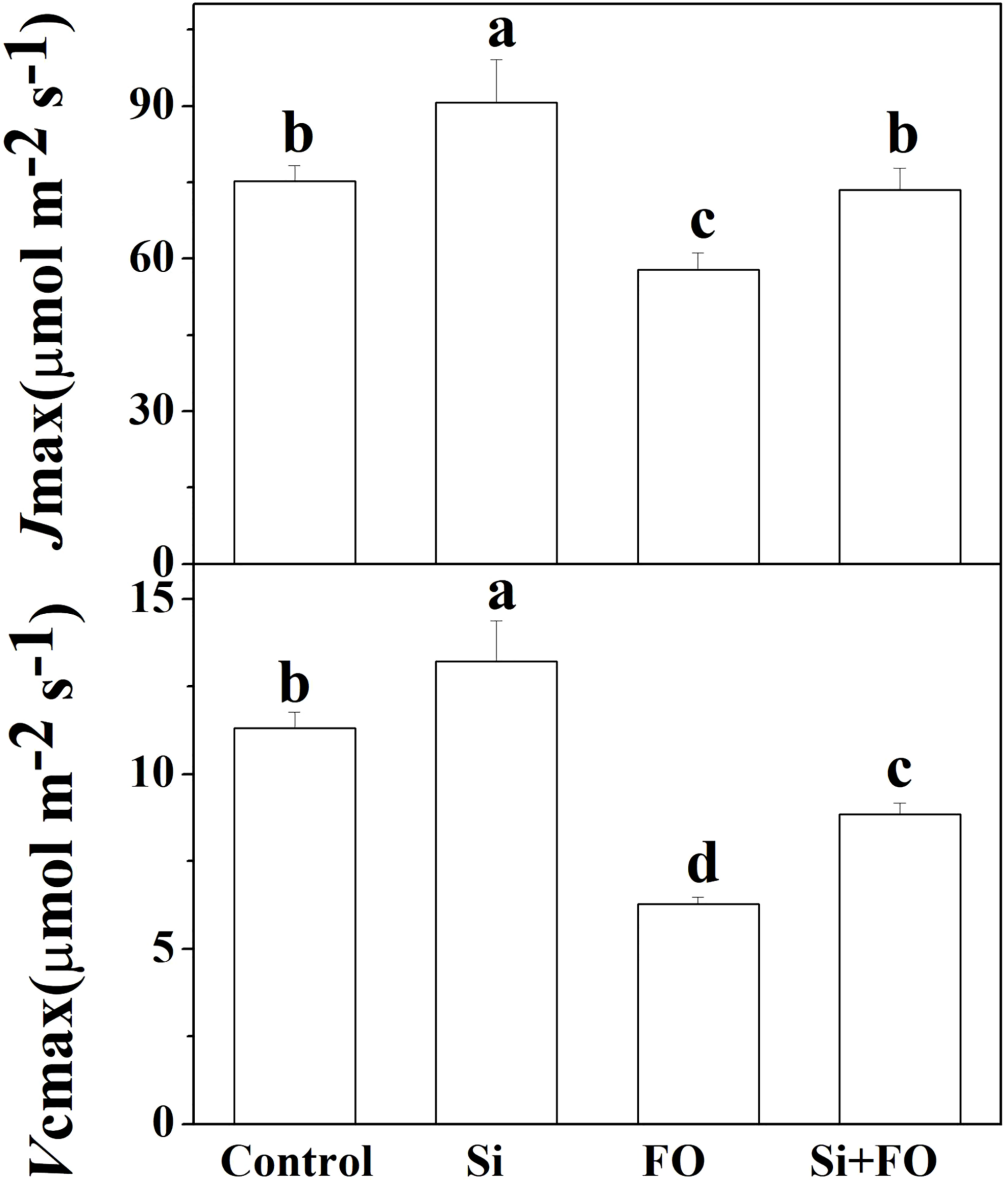
Effects of exogenous silicon application on *J*max and *V*cmax of cucumber leaves. Means denoted by the different lowercase letters are significantly different according to Tukey’s test (*P* ≤ 0.05); the mean represents the average of three replicates ± standard deviation (SD). Control: plants were irrigated with sterilized water; Si: plants were irrigated with exogenous silicon (3 mmol·l^-1^ Si); FO: plants were only inoculated with *Fusarium*; Si+FO: plants were irrigated with exogenous silicon followed by inoculation with *Fusarium.*.

Compared with the control, *V*cmax and *J*max in Si treatment increased by 16.82% and 20.56%, respectively. However, *V*cmax and *J*max in cucumber leaves decreased significantly after inoculation with *Fusarium* for 14 days. Meanwhile, *V*cmax and *J*max in plants pretreated with Si and then inoculated with *Fusarium* wilt were significantly increased by 40.97% and 27.07% compared with the cucumber plants inoculated with *Fusarium* only (**Figure 6**).

### Effects of exogenous silicon application on activities of RuBisCO, FBPase, and GAPDH in cucumber leaves

**Figure 7:**
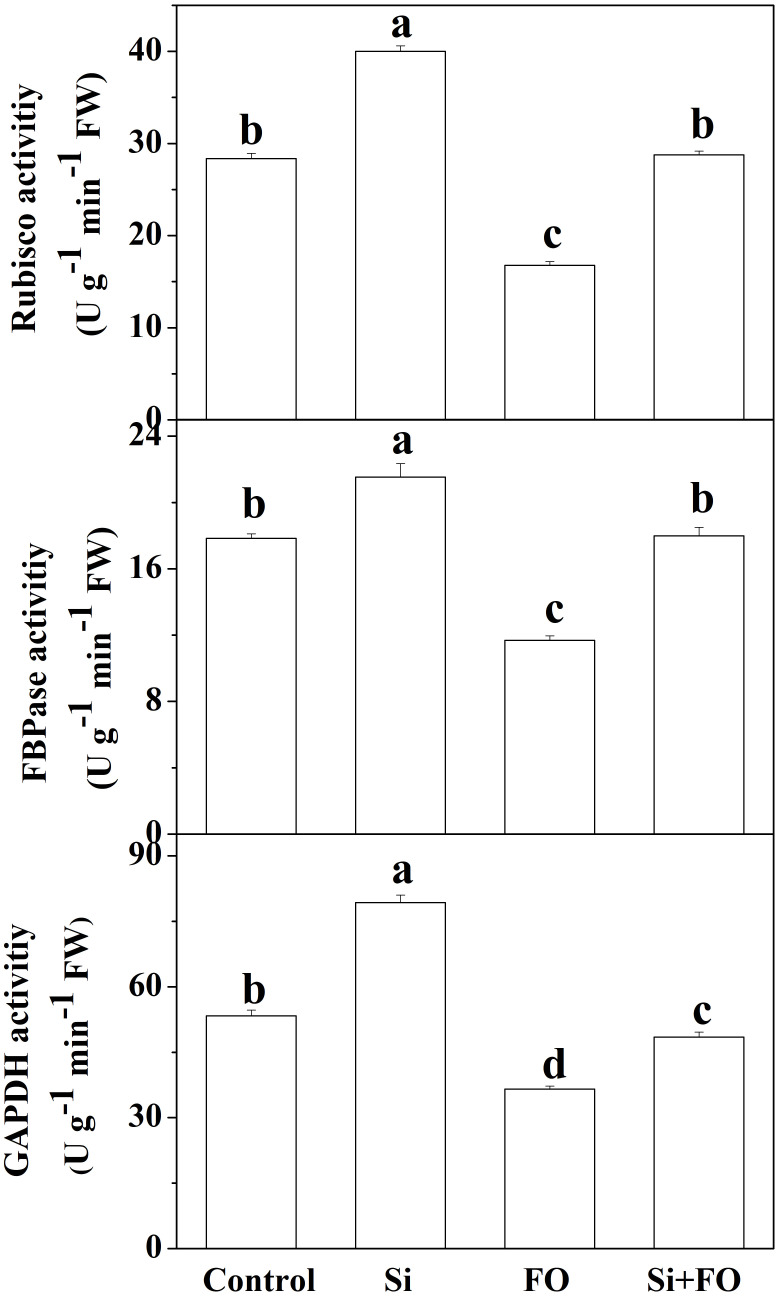
Effects of exogenous silicon application on activities of Rubisco, FBPase, and GAPDH in cucumber leaves. Means denoted by the different lowercase letters are significantly different according to Tukey’s test (*P* ≤ 0.05); the mean represents the average of three replicates ± standard deviation (SD). Control: plants were irrigated with sterilized water; Si: plants were irrigated with exogenous silicon (3 mmol·l^-1^ Si); FO: plants were only inoculated with *Fusarium*; Si+FO: plants were irrigated with exogenous silicon followed by inoculation with *Fusarium*.

Exogenous Si treatment induced the activities of three photosynthetic enzymes, namely, RuBisCO, FBPase, and GAPDH. However, inoculation with FO decreased the activities of RuBisCO, FBPase, and GAPDH. Furthermore, the activities of RuBisCO, FBPase, and GAPDH of the plants pretreated with Si and then inoculated with *Fusarium* significantly increased by 71.51%, 53.96%, and 32.55%, respectively, compared with the plants inoculated with *Fusarium* only (**Figure 7**).

### The effect of exogenous silicon on the expression of Calvin cycle-related genes in cucumber leaves

**Figure 8:**
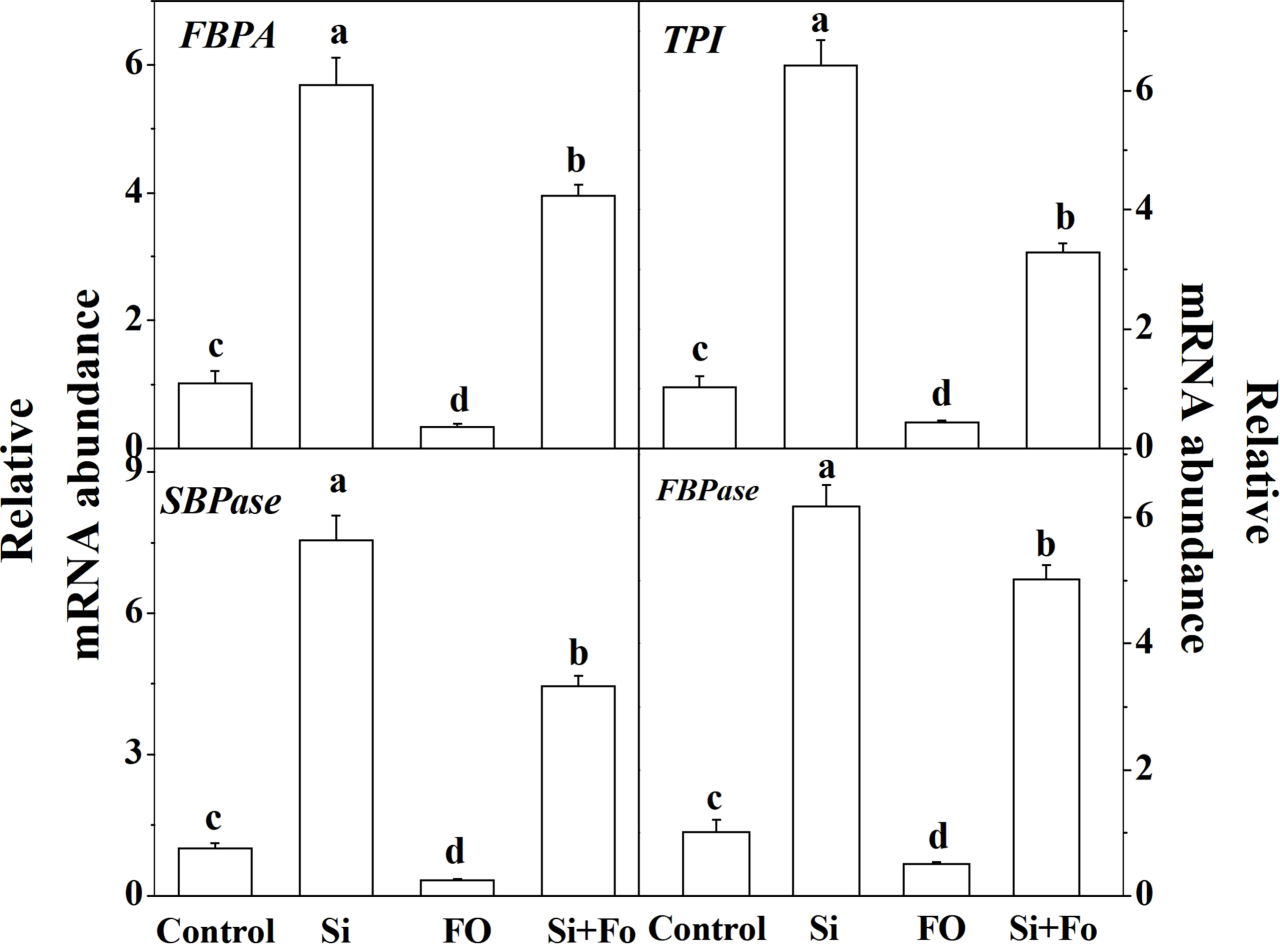
Effect of exogenous silicon on the expression of genes involved in the Calvin cycle in tomato leaves. Means denoted by the different lowercase letters are significantly different according to Tukey’s test (*P* ≤ 0.05); the mean represents the average of three replicates ± standard deviation (SD). Control: plants were irrigated with sterilized water; Si: plants were irrigated with exogenous silicon (3 mmol·l^-1^ Si); FO: plants were only inoculated with *Fusarium*; Si+FO: plants were irrigated with exogenous silicon followed by inoculation with *Fusarium.*.

To further study the role of exogenous silicon in photosynthesis, the expression of four genes involved in RuBP regeneration was determined. As shown in **Figure 8**, the expression of *FBPA*, *TPI*, *SBPase*, and *FBPase* of the plants pretreated with Si increased significantly compared with that of the control. On the contrary, the expression of *FBPA*, *TPI*, *SBPase*, and *FBPase* of the treatments inoculated with Fo decreased by 66.78%, 57.83%, 68.16%, and 49.59%, respectively, compared with that of the control. Meanwhile, the expression of *FBPA*, *TPI*, *SBPase*, and *FBPase* of the plants pretreated with Si and then inoculated with *Fusarium* significantly increased 10.61, 6.60, 12.77, and 8.84 times, respectively, compared with the plants inoculated with *Fusarium* only (**Figure 8**).

### Exogenous silicon application alleviates the stomatal closure of cucumber caused by *F. oxysporum*

**Figure 9:**
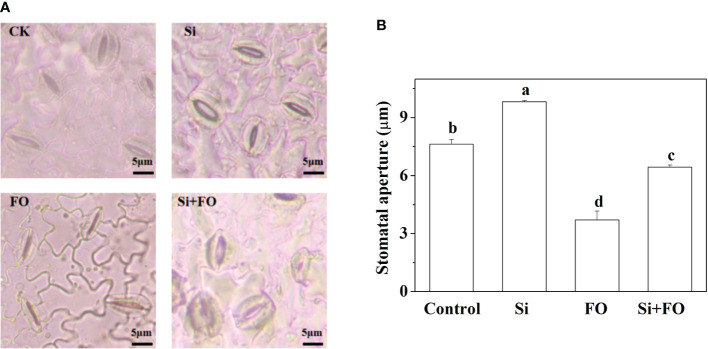
Effects of exogenous silicon application on stomatal movement in the lower epidermis of cucumber leaves. **(A)** Microscopic photographs of stomata in the lower epidermis, and **(B)** stomatal aperture as influenced by different treatments. Means denoted by the different lowercase letters are significantly different according to Tukey’s test (*P* ≤ 0.05). CK or Control: plants were irrigated with sterilized water; Si: plants were irrigated with exogenous silicon (3 mmol·l^-1^ Si); FO: plants were only inoculated with *Fusarium*; Si+FO: plants were irrigated with exogenous silicon followed by inoculation with *Fusarium*.

The stomatal opening of cucumber plants pretreated with Si was significantly higher than in other treatments. The stomata of the abaxial epidermis of cucumber leaves are almost completely closed at 14 days after inoculation with *F. oxysporum*. However, Si application alleviated the stomatal closure caused by *F. oxysporum*, and the stomatal opening was 42.39% higher than that of the plants inoculated with *F. oxysporum* only (**Figure 9**).

### Effects of exogenous silicon application on endogenous silicon content in cucumber

**Figure 10:**
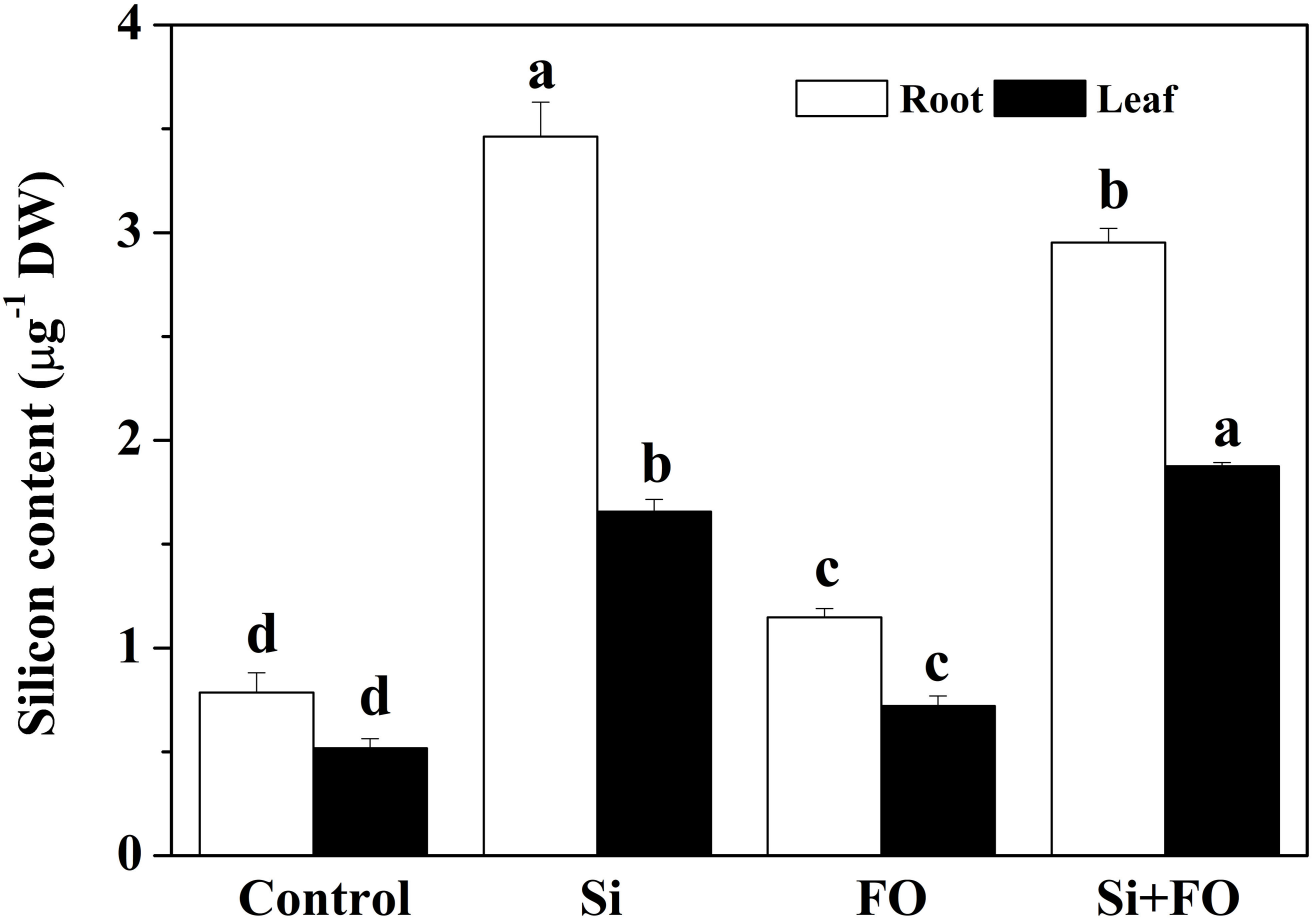
Effects of exogenous silicon application on endogenous silicon content in cucumber plants. Means denoted by the different lowercase letters are significantly different according to Tukey’s test (*P* ≤ 0.05); the mean represents the average of three replicates ± standard deviation (SD). Control: plants were irrigated with sterilized water; Si: plants were irrigated with exogenous silicon (3 mmol·l^-1^ Si); FO: plants were only inoculated with *Fusarium*; Si+FO: plants were irrigated with exogenous silicon followed by inoculation with *Fusarium.*.

The contents of endogenous Si in roots and leaves of cucumber plants pretreated with Si were 4.41 times and 3.21 times higher than those of the control, respectively, which indicated that cucumber roots can absorb Si from soil and transport it to leaves. The content of endogenous Si in roots and leaves in Fo treatment was 46.06% and 39.46%, respectively, higher than those in the control. Compared with Fo treatment, the content of endogenous Si in roots and leaves of Si+Fo treatment increased by 3.756 times and 3.63 times, respectively (**Figure 10**).

## Discussion

The exogenous application of Si has demonstrated an efficient ability in reducing both soil and airborne fungal diseases in a wide variety of crops (Kaushik and Saini, 2019; Ahammed and Yang, 2021). Silicon induces disease resistance *via* two key mechanisms. Firstly, the deposition of Si around the cell wall fosters mechanical protection which prevents pathogen penetration. In addition, Si forms complexes with organic compounds in the epidermal cell wall which also strengthen the cell wall mechanically (Mvondo-She et al., 2021). Secondly, Si induces systemic resistance by improving the upregulation of genes involved in metabolism, signal transduction, defense, and stress response against different plant pathogens (Kurabachew et al., 2013). Moreover, silicon may interact with key plant stress signal systems and eventually induce resistance to pathogens. These mechanisms have been revealed in multiple plant species including pepper and melon (Pozo et al., 2015), cucumber (Silva et al., 2020), onion and garlic (Elshahawy et al., 2021), tomato (Madany et al., 2020), and carrot (Ahamad and Siddiqui, 2021). In the present study, we found that Si alleviated oxidative stress and the inhibition of photosynthesis in cucumber caused by the *Fusarium* wilt by activating the antioxidant system, thereby improving the photosynthetic capacity of cucumber leaves.

*Fusarium* wilt of cucumber is one of the three major soil-borne diseases, and its incidence rate is as high as 70%, which reduces the yield of cucumber by 15% to 50%, thus severely restricting the production of greenhouse cucumber (Raza et al., 2017). Silicon application can inhibit fungal diseases by increasing Si accumulation in silicified cells of the leaves of gramineous plants, forming a “keratin-silicon double layer”, or inducing plants to produce phytoalexins and antitoxins (Camargo et al., 2021). In this study, the optimum Si concentration (3 mmol/l) was selected from five different concentrations of Si based on their inhibitory effects on *Fusarium* growth (**Figure 1**). Subsequent experiments show that exogenous silicon can significantly increase the growth of cucumber plants infected by *F. oxysporum*, which provides a theoretical basis for the application of Si in the control of *Fusarium* wilt in cucumber.

Photosynthesis-related factors play a crucial role in plant metabolism and are involved in defense against pathogens (Letousey et al., 2010). Fungal diseases can inhibit photosynthetic capacity by changing the chloroplast structure and reducing the chlorophyll content and photosynthesis-related enzyme activity (Fernandez-Martinez et al., 2013). In the early stage of infection, *Fusarium* wilt induces decreases in the light-saturated rate of CO_2_ assimilation, which are accompanied by decreases in the maximum carboxylation rate and the capacity for RuBP regeneration as well as increases in stomatal limitation, in the absence of any significant photodamage to photosystem II (Nogues et al., 2002). *F. oxysporum* infection also induces a decrease in net photosynthetic rate in the early stage in banana plants, which is mainly resulted from stomatal limitation, and the damage to chloroplasts contributes to the reduction in the photosynthetic capacity in the later stages of infection (Dong et al., 2016). The supply of Si to rice plants played a central role in decreasing leaf scald symptoms and enhanced the maximum electron transport rate and RuBisCO activity (Pereira et al., 2020).

The expression of four Calvin cycle-related genes encoding proteins such as FBPA, TPI, SBPase, and FBPase was repressed in leaves of *Fusarium*-inoculated cucumber plants (**Figure 8**). The repression of the gene expression was in agreement with the reduction in the activity of Rubisco, FBPas, and GAPDH. Notably, SBP and Rubisco are known to have an essential role in the control of photosynthetic carbon fixation (Letousey et al., 2010), suggesting that a metabolic alteration in photosynthetic reaction occurred in leaves due to *Fusarium* inoculation in cucumber plants. However, Si application significantly increased the *P*n, *G*s, Tr, *V*cmax, and *J*max activities of RuBisCO, FBPase, and GAPDH and the expression of related genes such as *FBPA*, *TPI*, *SBPase*, and *FBPase* in cucumber leaves and reduced Ci. It indicated that the decline in photosynthetic capacity caused by *F. oxysporum* was not only related to the decrease in stomatal conductance and the obstruction of CO_2_ supply but also related to the non-stomatal factors that led to the decrease in photosynthetic rate caused by the decrease in photosynthetic activity of mesophyll cells (Ahammed et al., 2020). Moreover, non-stomatal limitations of photosynthesis often involve disruptions in metabolic pathways of photosynthesis and are common in response to biotic stress (Letousey et al., 2010). In view of the close relationship between the disease resistance effect of Si and the photosynthetic physiology of plant leaves, the photosynthetic rate of leaves can be used as a reference index for screening the Si effect.

Silicon application has been shown to have a dose-dependent effect on enhancing plant resistance to diverse pathogens (Kaushik and Saini, 2019; Ahammed and Yang, 2021). The shoot Si content was negatively correlated with the severity of red crown rot caused by *Calonectria ilicicola* in soybean (Win et al., 2021). Moreover, aboveground biomass and seed yield at harvest increased with increasing Si concentration in soil (0.0–3.0 g Na_2_SiO_3_ kg^-1^ soil). However, a certain high dose of Si (6.0 g Na_2_SiO_3_ kg^-1^ soil) could reduce seed yield (Win et al., 2021). There were also some opposite reports. Silicon application significantly reduced the incidence of white rot and improved the growth of onion and garlic plants (Elshahawy et al., 2021). However, there were no significant differences between some treatments at 0.1%, 0.2%, and 0.3% of silicon and silicate salts. In the present study, the silicon concentration of 3 mmol/l had the best effect on the improvement of photosynthesis, but when the silicon concentration increased to 5 mmol/l, the effect was largely decreased. This might be correlated with the alterations of the homeostatic network of mineral elements (Etesami and Jeong, 2018).

More evidence showed that Si may participate in the metabolic process to enhance plant disease resistance through a series of physiological and biochemical reactions and signal transduction (Ahammed and Yang, 2021). Enhanced resistance is achieved by activating host defense genes and inducing the production of a series of low molecular weight metabolites. Firstly, Si can enhance the activities of protective enzymes to improve disease resistance (Etesami and Jeong, 2018). Silicon was associated with increased activity of superoxide dismutase in shoots and roots and increased tissue concentrations of phenolics, proline, and antioxidants, but reduced levels of H_2_O_2_ (Moradtalab et al., 2018). Secondly, Si can improve host resistance to diseases by inducing the production of some secondary metabolites or antibacterial substances such as phytoalexin, lignin, phenols, and pathogen-related proteins (Ahammed and Yang, 2021). Notably, the induction of antioxidant defense is often associated with ROS generation under stress conditions, particularly in response to pathogen attack. In the present study, Si application (3 mmol/l) significantly increased the antioxidant enzyme activities and decreased H_2_O_2_, $O_{2}^{\cdot -}$ , and MDA contents in leaves of cucumber under the stress caused by *F. oxysporum* inoculation, thus mitigating oxidative damage.

**Figure 11:**
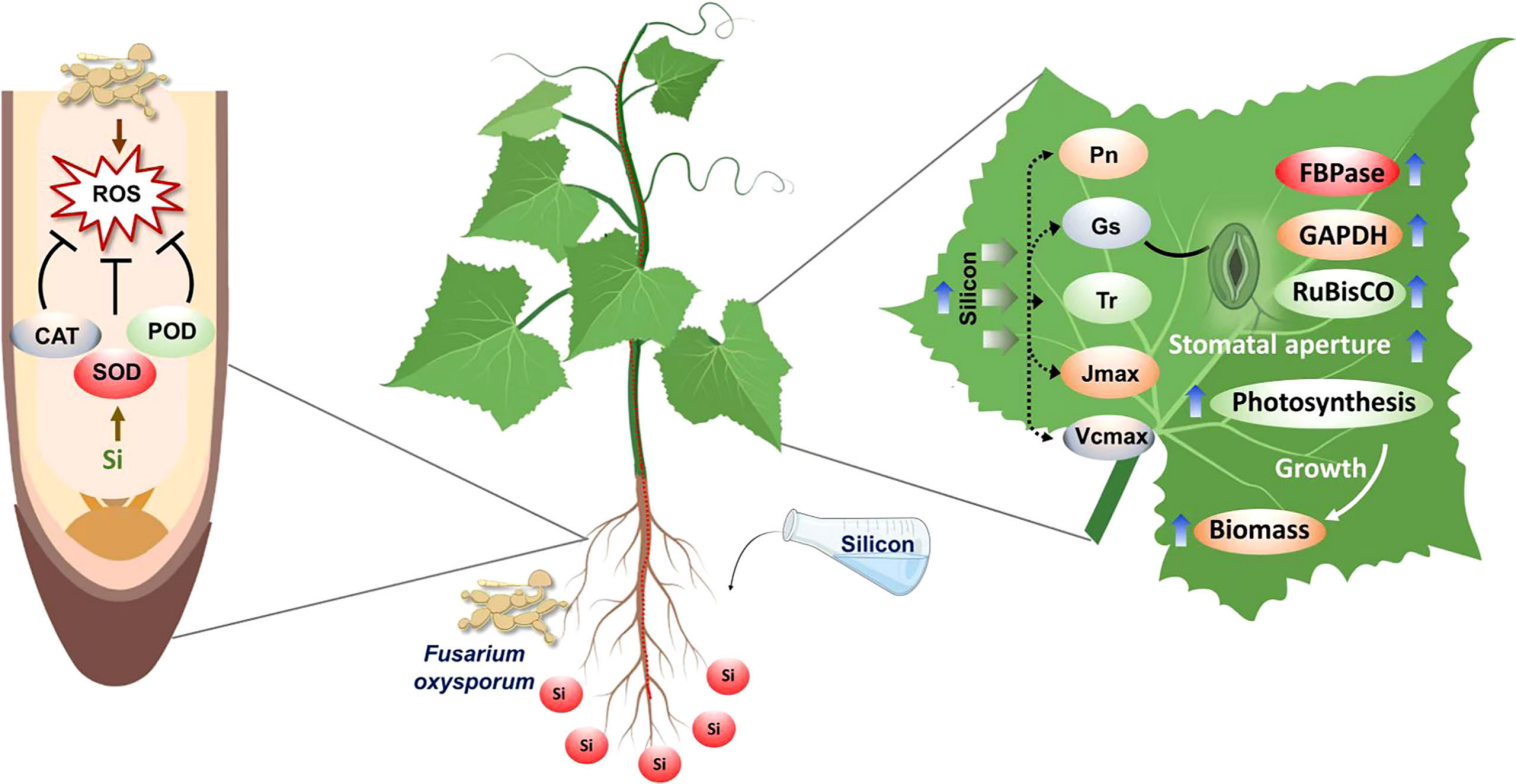
A working model depicting the mechanism of silicon-mediated alleviation of Fusarium wilt stress as revealed in the present study.

In conclusion, in the current study, we screened out effective concentrations of Si that could significantly inhibit the mycelial growth of Fo and revealed the plant defense mechanisms triggered by exogenous silicon in response to Fo inoculation in cucumber plants. Exogenous Si (3 mmol l^-1^) application combined with Fo inoculation substantially boosted plant growth, root activity, and antioxidant enzyme activity but reduced ROS accumulation and lipid peroxidation compared to Fo alone (**Figure 11**). Silicon treatment also increased photosynthetic capacity by alleviating both stomatal and non-stomatal limitations, particularly by attenuating stomatal closure and increasing activities of CO_2_ assimilation-related enzymes such as RuBisCO. Taken together, our study suggests that exogenous silicon can increase cucumber resistance to *Fusarium* wilt by increasing antioxidant defense, photosynthetic capacity, and stomatal opening. However, further studies are still required to better understand how silicon regulates plant signal transduction to enhance plant resistance against pathogenic fungi.

## Data availability statement

The datasets presented in this study can be found in online repositories. The names of the repository/repositories and accession number(s) can be found in the article/**Supplementary Material**.

## Author contributions

SS: methodology, formal analysis, writing—review and editing. ZY: methodology, formal analysis, investigation, writing—original draft. ZS: formal analysis, investigation. NW: formal analysis, investigation. NG: formal analysis, investigation. JN: formal analysis, investigation. AL: conceptualization, supervision, funding acquisition. BB: supervision. GJA: conceptualization, writing—review and editing, supervision, funding acquisition. SC: conceptualization, writing—original draft, writing—review and editing, supervision, funding acquisition, project administration. All authors contributed to the article and approved the submitted version.

## Funding

This research was supported by the National Key Research and Development Program of China (2018YFD1000800), National Natural Science Foundation of China (31872092, 31872157, 31950410555), Natural Science Foundation of Henan (202300410152), Innovative Research Team (in Science and Technology) in University of Henan Province (23IRTSTHN024), Scientific and Technological Research Project of Henan (222102110078), Luoyang Rural Revitalization Project (2101101A), and Ministry of Science and Technology of the People’s Republic of China (DL2022026004L, QNJ2021026001).

## Conflict of interest

The authors declare that the research was conducted in the absence of any commercial or financial relationships that could be construed as a potential conflict of interest.

## Publisher’s note

All claims expressed in this article are solely those of the authors and do not necessarily represent those of their affiliated organizations, or those of the publisher, the editors and the reviewers. Any product that may be evaluated in this article, or claim that may be made by its manufacturer, is not guaranteed or endorsed by the publisher.
