# Supplementary material for: Silicon enhances plant resistance to Fusarium wilt by promoting antioxidant potential and photosynthetic capacity in cucumber (Cucumis sativus L.)
Source: Front Plant Sci. 2022 Oct 13;13:1011859. doi: 10.3389/fpls.2022.1011859 (PMC9608603; doi:10.3389/fpls.2022.1011859)
Supplement: Supplementary file 1 [file Table_1.docx]

**Supplementary Material**

**Table 1 Primers used for the gene expression assay in cucumber leaves by real-time qPCR**

| Gene | Accession No. | Full name of gene | Forward primer | Reverse primer | Product size (bp) |
| --- | --- | --- | --- | --- | --- |
| *FBPA* | GT564642.1 | Fructose-1,6-bisphosphate aldolase | 5’-GCACAGAAAGTTTGGGCAGA-3’ | 5’-TATGCTTCCGGCTCGTATGT-3’ | 212 |
| *TPI* | XM_004143068.3 | Triosephosphate | 5’-TCTCCAGCTGGGACAATGTT-3’ | 5’-ATATCCACGAACTCCGGCTT-3’ | 248 |
| *SBPase* | NM_001280729.1 | Sedoheptulose-1,7-bisphosphatase | 5’-TGCTCTGAAGAAGTGCCAGA-3’ | 5’-GTAAACTCCCATGGCAGCAG-3’ | 178 |
| *FBPase* | XM_004137013.3 | Fructose 1,6-bisphosphatase | 5’-TTCGACTGGCCAGAAGGATT-3’ | 5’-GCTTACCTGGCTGCAATGAA-3’ | 245 |
| *actin* | XM_004136807.3 |  | 5’-AGCAACTGGGATGACATGGA-3’ | 5’-CCCTCGTATATGGGCACTGT-3’ | 226 |
